# Adipose tissue-derived metabolite risk scores and risk for type 2 diabetes in South Asians

**DOI:** 10.1038/s41366-023-01457-4

**Published:** 2024-01-20

**Authors:** Meghana D. Gadgil, Jing Cheng, David M. Herrington, Namratha R. Kandula, Alka M. Kanaya

**Affiliations:** 1 Division of General Internal Medicine, Department of Medicine, University of California, San Francisco School of Medicine, 1545 Divisadero Street, Suite 320, San Francisco, CA 94143 USA; 2 Department of Preventive and Restorative Dentistry, University of California, San Francisco School of Dentistry, 707 Parnassus Ave, #1026, San Francisco, CA 94143 USA; 3 Section on Cardiovascular Medicine, Department of Internal Medicine, Wake Forest School of Medicine; Medical Center Boulevard, Winston-Salem, NC 27157 USA; 4 Division of General Internal Medicine, Department of Medicine, Northwestern University Feinberg School of Medicine, 750 N. Lakeshore Dr. 6h Floor, Chicago, IL 60611 USA; 5 Department of Epidemiology and Biostatistics, University of California, San Francisco School of Medicine, 550 16th Street, Second Floor, San Francisco, CA 94158 USA

**Keywords:** Type 2 diabetes, Risk factors

## Abstract

**Background:**

South Asians are at higher risk for type 2 diabetes (T2D) than many other race/ethnic groups. Ectopic adiposity, specifically hepatic steatosis and visceral fat may partially explain this. Our objective was to derive metabolite risk scores for ectopic adiposity and assess associations with incident T2D in South Asians.

**Methods:**

We examined 550 participants in the Mediators of Atherosclerosis in South Asians Living in America (MASALA) cohort study aged 40–84 years without known cardiovascular disease or T2D and with metabolomic data. Computed tomography scans at baseline assessed hepatic attenuation and visceral fat area, and fasting serum specimens at baseline and after 5 years assessed T2D. LC-MS-based untargeted metabolomic analysis was performed followed by targeted integration and reporting of known signals. Elastic net regularized linear regression analyses was used to derive risk scores for hepatic steatosis and visceral fat using weighted coefficients. Logistic regression models associated metabolite risk score and incident T2D, adjusting for age, gender, study site, BMI, physical activity, diet quality, energy intake and use of cholesterol-lowering medication.

**Results:**

Average age of participants was 55 years, 36% women with an average body mass index (BMI) of 25 kg/m^2^ and 6% prevalence of hepatic steatosis, with 47 cases of incident T2D at 5 years. There were 445 metabolites of known identity. Of these, 313 metabolites were included in the MET-Visc score and 267 in the MET-Liver score. In most fully adjusted models, MET-Liver (OR 2.04 [95% CI 1.38, 3.03]) and MET-Visc (OR 2.80 [1.75, 4.46]) were associated with higher odds of T2D. These associations remained significant after adjustment for measured adiposity.

**Conclusions:**

Metabolite risk scores for intrahepatic fat and visceral fat were strongly related to incident T2D independent of measured adiposity. Use of these biomarkers to target risk stratification may help capture pre-clinical metabolic abnormalities.

## Introduction

Obesity measured by body mass index (BMI) criteria has well-established associations with cardiometabolic disease but has limitations that are increasingly being recognized [1–3]. Across the BMI spectrum, the presence of ectopic adiposity has been associated with a “metabolically unhealthy” phenotype, independent of BMI [4–6]. Ectopic adiposity encompasses visceral fat present around abdominal organs, hepatic steatosis (intrahepatic fat), pericardial fat, and intermuscular fat, and is an emerging risk factor for cardiometabolic disease [7]. As the prevalence of diabetes varies substantially by race/ethnicity [8] independent of BMI, the presence of ectopic adiposity may be a stronger risk factor for diabetes and cardiovascular disease than BMI alone.

South Asians have a higher risk for cardiometabolic disease than many other race/ethnic groups, at lower BMI. In a group of U.S. South Asians in the Mediators of Atherosclerosis in South Asians Living in America (MASALA) study, there was a higher age-adjusted prevalence of diabetes at 23% when compared with other racial and ethnic groups. In this study, liver and pericardial fat were higher despite a lower BMI and waist circumference than a non-Hispanic White population [9]. The prevalence and severity of hepatic steatosis was found to be greater in South Asians in the MASALA study as compared to Black, White, Chinese and Hispanic American participants in the MESA study [10], while visceral fat area was comparable.

Circulating metabolites and lipids are small molecules that result from cellular processes, and characterization of a pattern of these compounds may allow for identification of metabolically active adiposity. An investigation in a cohort of individuals from India, the Cardiometabolic Risk Reduction in South Asia (CARRS) Study, has characterized general and central-obesity associated lipids in South Asians in the diaspora [11]. Total cholesterol in HDL was associated with both general obesity and with a lower odds of type 2 diabetes, while 15 nonoverlapping metabolites were associated with central obesity, of which 10 were prospectively associated with higher type 2 diabetes risk [11]. Separately, recent work has shown evidence of BMI-independent heritability of adipose tissue depots [12] and genetic predisposition of high waist-hip ratio to cardiometabolic outcomes [13]. Several prior studies in MASALA have shown less favorable body composition profiles in South Asians compared with people of other races/ethnicities, however they do not completely explain the excess risk for diabetes [14].

As metabolically active adiposity is a risk for a variety of cardiometabolic diseases, our goal is to assess BMI-independent measures of adiposity, their representative metabolites and lipids and their prospective associations with metabolic disease in South Asians. Specifically, we aim to define a metabolomic signature of visceral adipose tissue area and liver attenuation, and to associate these signatures with incident type 2 diabetes.

## Methods

### Participants

Data were from South Asian individuals who participated in the MASALA community-based cohort study and had metabolomic and computed tomography data from Exam 1. The detailed methods have been described elsewhere [15]. MASALA is a prospective cohort study which enrolled community-dwelling individuals living in the San Francisco Bay Area and the greater Chicago areas from 2010 to 2013. Participants self-identified as being of South Asian ancestry (one out of four grandparents were from India, Pakistan, Bangladesh, Sri Lanka or Nepal) and were aged 40–84 years and without known cardiovascular disease. After approximately 4.8 years of follow-up, 749 (83%) participants from the entire cohort returned to complete Exam 2. Those on nitroglycerin, with active cancer, with impaired cognitive ability, a life expectancy less than five years, who lived in a nursing home, or who had plans to relocate were excluded. Participants with self-reported cirrhosis (*n* = 1) and alcohol consumption of >7 drinks/week (*n* = 41) were excluded to restrict the analysis sample to those with likely steatotic liver disease. We then excluded 127 participants without LC-MS metabolomics measurements, 134 participants with prevalent T2D and 33 participants without visceral or intrahepatic fat measurements. For this analysis, we used data from a subset of 550 participants who did not have diabetes at baseline and had metabolomics data. (Supplementary Fig. 1)

The University of California, San Francisco and Northwestern University Institutional Review Board approved the study protocol and all study participants provided written informed consent.

### Demographic and clinical data

Each participant underwent in-person interviews to determine age, gender, medical history, physical activity (MET-minutes/week), diet quality as defined by the Alternative Healthy Eating Index-2010 (AHEI-2010), energy intake (kcal/day), smoking status and alcohol intake.

### Metabolic profiling by UPLC-MS

A total of 754 serum samples obtained at Exam 1 (2010–2013) were analyzed by ultra‐performance liquid chromatography mass spectrometry (UPLC-MS) using analytical and quality control procedures described in detail elsewhere [16, 17]. Sample analysis was performed in a way designed to be orthogonal to clinical and demographic data. For quality control assessment and data pre-processing, a study reference sample was prepared by pooling equal parts of each study sample.

Serum samples were prepared and analyzed using UPLC-MS as previously published [16, 17]. In brief, 50 μL aliquots were taken from each sample, diluted 1:1 with ultrapure water for lipid profiling and 1:1.4 for small molecule profiling. Protein was removed by addition of organic solvent to the diluted sample (four volumes isopropanol per volume of diluted sample for lipidomic profiling and three volumes of acetonitrile per volume of diluted sample for small molecule profiling) followed by mixing and centrifugation to yield a homogenous supernatant. Aliquot sets of prepared samples were subjected to chromatographic separation using an ACQUITY UPLC (Waters Corp., Milford, MA, USA) system. Lipidomic profiling was performed using reversed-phase chromatography (RPC) with a 2.1 × 100 mm Acquity BEH C8 column maintained at 55 °C. The chromatographic separation was performed using a binary mobile phase system consisting of (A) a 50:25:25 mixture of H_2_O:ACN:IPA with 5 mm ammonium acetate, 0.05% acetic acid, and 20 µM phosphoric acid and (B) 50:50 ACN:IPA with 5 mm ammonium acetate, 0.05% acetic acid. Polar metabolite profiling was completed using hydrophilic interaction liquid chromatography (HILIC) with a 2.1 × 150 mm Acquity BEH HILIC column maintained at 40 °C. The chromatographic separation used a binary mobile phase system consisting of (A) acetonitrile with 0.1% formic acid and (B) 20 mM ammonium formate in water with 0.1% formic acid. Both separation types were coupled to high resolution mass spectrometry (Xevo G2‐S TOF mass spectrometers, Waters Corp., Manchester, UK) via a Z‐spray electrospray ionization source. The lipidomic profiling assay was conducted in both positive and negative ion modes (generating Lipid RPC+ and Lipid RPC- datasets), while the HILIC assay was performed in the positive ion mode only (generating the HILIC+ dataset). A SR sample was acquired every 10 study samples throughout the analysis. In addition, a dilution series was created from the SR and analyzed immediately prior to and after the study sample analysis for use in signal filtering as described previously [16].

Raw data was converted to the mzML open source format and signals below an absolute intensity threshold of 100 counts were removed using the MSConvert tool in ProteoWizard [18]. Metabolite signal extraction was performed using PeakPantheR, an open-source package to detect, integrate and report pre-defined and annotated lipids and metabolites from an in-house database [19]. Elimination of potential run-order effects and filtering of the extracted metabolites was performed using the nPYc-Toolbox, an open-source package for data pre-processing [20]. Only those measured with high accuracy (relative coefficient of variance in SR samples less than 20%) and high precision (correlation to dilution in SR dilution series greater than 0.8) were retained and put forward for biological analysis. Of the 754 total study samples, 32 were not included in our analysis due to insufficient sample volume and five were excluded due to missed injection in the HILIC assay.

#### Cardiometabolic factors measured at baseline

Weight was determined using a digital scale, height with a stadiometer, and waist circumference using a measuring tape halfway between the lower ribs and the anterior superior iliac spine, at the site of greatest circumference. Hip circumference was measured at the maximum girth of the buttocks. Blood samples were obtained after a requested 12-h fast. Fasting plasma glucose was measured using the hexokinase method (Quest diagnostics, San Jose, CA). Type 2 diabetes was defined as a fasting glucose ≥126 mg/dl or use of a glucose-lowering medication.

#### Metabolic measures at Exam 2

We assessed incident diabetes and fasting plasma glucose at Exam 2 with the methods described above. The change in glucose was calculated as the difference between fasting glucose measurement (mg/dL) at Exam 2 and at Exam 1. There were 50 cases of incident diabetes at Exam 2.

### Body composition measures

Non-contrast cardiac CT images were obtained to quantify pericardial fat and hepatic attenuation using a cardiac-gated CT scanner: at UCSF, a Phillips 16D scanner or a Toshiba MSD Aquilion 64 and at NWU, a Siemens Sensation Cardiac 64 Scanner (Siemens Medical Solutions, Malvern, PA, USA) was used. The same reading center staff under the supervision of Dr Jeffrey Carr performed all measurements of pericardial fat volume and hepatic attenuation. The CT scan range encompassed the entire heart and provided information on 45 mm of adipose tissue encasing the proximal coronary arteries. We first defined the 45 mm z axis volume containing the proximal coronary arteries. The technician follows a set of regions of interest pertaining to subcutaneous and pericardial fat within the 45 mm volume along with regions in the calibration phantom to calculate the range of Hounsfield units for adipose tissue. The technician segments the heart from the thorax by removing tissues beyond the lung using a deformable model-based edge detection method such as active contours or live wires to detect the boundary between the lung and fat around the heart.19–21 CT images for hepatic attenuation were also interrogated using the MIPAV software at vertebral level T12-L1. Nine regions of interest within homogenous portions of the liver at two levels were read, avoiding any vascular structures or other liver pathology. Assessment of hepatic attenuation and ectopic fat was done with non-contrast computed tomography (CT) images obtained at Exam 1 with electron-beam or multidetector CT scanners as previously described [10]. Non-contrast cardiac CT images were used to quantify hepatic attenuation. There were nine regions of interest read within homogenous portions of the liver at two levels. Lower values of hepatic attenuation measured in Hounsfield Units (HU) correspond to greater quantity of intrahepatic fat; to improve the interpretation and comparability of results, we calculated the inverse of hepatic attenuation values by multiplying the measured values by −1. Steatotic liver disase was defined as a dichotomous variable with hepatic fat attenuation <40 HU.

A trained CT technician obtained a lateral scout image of the abdomen to establish position between the L4 and L5 vertebrae. Medical Image Processing, Analysis, and Visualization (MIPAV) software (Center for Information Technology and National Institutes of Health 1999) was used to interrogate CT images at vertebral levels L4-L5 for the visceral fat, intermuscular fat and subcutaneous fat measurements. The subcutaneous tissue compartment included tissue outside the visceral cavity but within the body contour, and visceral fat was defined as fat with the appropriate HU within the visceral cavity.

### Statistical methods

Before modeling, relative abundance of metabolites were log-transformed to reduce the potential for outliers to influence the model. To adjust for unreliable parameter estimates that may occur when using multiple regression models in the setting of multicollinearity, we performed an elastic net regularized regression model to evaluate all metabolites (446 annotated LC-MS metabolites) for their associations with each body composition outcome. The elastic-net model allowed for a penalized linear regression on all biomarkers simultaneously to identify the metabolites most highly associated with each outcome. Optimal parameters for the penalty value (α) and the regularization penalty (λ) were determined by 10-fold cross-validation.

Data in the full dataset were randomly assigned to one of two equal sized datasets (“training” and “testing”). Model performance was judged based on root mean square error, with the model chosen minimizing mean cross-validated error. Optimization was completed using STATA’s “elasticnet” and postestimation commands for model prediction using 10-fold cross-validation. For the training set, we built an elastic net model with a penalty weight of alpha = 0.3. The shrinkage parameter lambda was optimized using a 10-fold cross-validation framework. From a total of 445 known metabolites in the original dataset, the elastic net model selected sets of metabolites significantly associated with each measure of adiposity (hepatic attenuation and visceral fat area). We then applied the trained model to the testing set to calculate predicted metabolite scores of inverse hepatic attenuation (MET-Liver) and visceral fat area (MET-Visc) for all participants. These metabolite profile scores were calculated as the weighted sum of the selected metabolites with weights equal to the elastic net regression coefficients [21].

For the analysis of incident diabetes, we used logistic regression models with robust standard errors to assess associations of continuous inverse hepatic attenuation, visceral fat area, MET-Liver and MET-Visc) with incident diabetes at five-year follow-up adjusting for age, gender, and study site (Model 1). We then further adjusted these logistic regression models for BMI, physical activity, diet quality, energy intake and use of lipid-lowering medication (Model 2) to incorporate covariates associated with T2D risk. Inclusion of hypertension or use of medications used to treated hypertension as covariates did not significantly change our point estimates and were therefore excluded from our analysis. As a sensitivity analysis, we stratified by glycemic status (normoglycemic and impaired fasting glucose at baseline).

The analysis was completed using STATA (version 16.1, 2021, College Station, TX, USA).

## Results

The analysis included 550 participants from the MASALA study with measurements of ectopic fat and LC-MS metabolomics data and without prevalent diabetes at enrollment. At Exam 1, average age was 55 years, the participants were 36% women with an average body mass index (BMI) of 25 kg/m^2^ and fasting glucose of 93 mg/dL. One-fifth of participants used lipid-lowering medications, and 1/3 had metabolic syndrome, as defined by a waist circumference of >94 cm in men and >80 cm in women. Half of participants had a family history of diabetes (Table 1).

**Table 1:** Clinical and demographic characteristics of the MASALA study population without prevalent diabetes at Exam 1.

**Demographic characteristics**	
Age (years)	55 (9)
Female *N* (%)	277 (36)
**Behavioral characteristics**	
Physical activity (MET-minutes/week) (Median, IQR)	990 (623)
Caloric intake (Kcal/day)	1673 (498)
AHEI-2010 Score	70 (7)
Smoking, never *N* (%)	477 (87)
Alcohol use, ever *N* (%)	168 (31)
**Medical history and Metabolic characteristics**	
Lipid-lowering medication use *N* (%)	105 (19)
Family history of diabetes, yes *N* (%)	263 (48)
Normoglycemic *N* (%)^a^	398 (72)
Metabolic syndrome *N* (%)^b^	159 (29)
Body Mass Index (kg/m^2^)	25 (4)
Fasting glucose (mg/dL)	93 (12)
Waist circumference (cm)	92 (10)
**Radiographic characteristics**	
Steatotic liver disease (HU < 40) (No, %)	35 (6)
Liver fat attenuation (HU)	57 (10)
Visceral fat area (cm^2^)	129 (52)
Subcutaneous fat area (cm^2^)	235 (89)
**Calculated adiposity**	
MET-Visc	133 (31)
MET-Liver	55 (7)

*n* = 550, Mean (SD) unless otherwise specified.

^a^Normoglycemic (fasting glucose <100 mgdL and not taking hypoglycemic medications).

^b^Metabolic syndrome definition (waist circumference ≥94 cm for men, ≥80 cm for women).

Using elastic net analyses, we identified metabolite profiles of intrahepatic fat and visceral fat area. Intrahepatic fat was represented by 267 metabolites and visceral fat area by 313 metabolites (Supplementary Table 1). Of those metabolites representing intrahepatic fat, nearly all were present in the group of metabolites characterizing visceral fat area.

In Model 1 analyses, with adjustment for age, gender, and study site, the odds of incident diabetes with a 1-standard deviation increase in intrahepatic fat was OR 1.64 [95% CI 1.25, 2.16]. In the most fully adjusted model, after additional adjustment for physical activity, BMI, caloric intake, AHEI-2010 Diet Quality Score, and use of lipid-lowering medications, there was a slight attenuation in the association, with OR 1.54 [1.13, 2.10] (*p* < 0.01). With use of the metabolite score for intrahepatic fat (MET-Liver), the odds of incident diabetes was OR 2.04 [95% CI 1.38, 3.02] (*p* < 0.01) in the most fully adjusted model. Similarly, the odds of incident diabetes was nearly 2-fold higher with one standard deviation increase in measured visceral fat area, OR 1.98 [95% CI 1.26, 3.11] in the most fully adjusted model. The odds of incident diabetes were OR 2.80 [1.75, 4.46] with the MET-Visc index. (Table 2) There were no interactions by gender found in any model.

**Table 2:** Odds of incident diabetes at 5 years by measured and predicted values for intrahepatic fat and visceral fat^a^.

	Odds^b^ of Incident Diabetes, Odds Ratio [95% CI]					
	Unadjusted	*P* value	Model 1^c^	*P* value	Model 2^d^	*P* value
	**Hepatic fat, measured by CT or metabolite score**					
Intrahepatic fat^a^	1.61 [1.23, 2.10]	4.48e−04	1.65 [1.25, 2.16]	3.26e−04	1.53 [1.13, 2.10]	6.71e−03
MET-Liver	1.91 [1.38, 2.65]	1.1e−04	2.04 [1.46, 2.86]	3.33e−05	2.04 [1.37, 3.03]	3.91e−04
	**Visceral fat, measured by CT or metabolite score**					
Visceral fat	1.76 [1.36, 2.27]	1.76e−05	1.86 [1.39, 2.48]	2.44e−05	1.98 [1.26, 3.11]	3.18e−03
MET-Visc	2.23 [1.52, 3.25]	3.46e−05	2.43 [1.67, 3.55]	4.25e−06	2.80 [1.75, 4.46]	1.53e−05
	**Metabolite-derived adiposity scores adjusted for CT-measured adiposity**					
MET-Liver, adjusted for Intrahepatic fat	–	–	–	–	1.87 [1.06, 3.27]	0.03
MET-Visc, adjusted for visceral fat	–	–	–	–	2.38 [1.34, 4.25]	3.20e−03
	**Measured visceral fat area adjusted for hepatic attenuation**					
Intrahepatic fat	–	–	–	–	1.36 [0.97, 1.92]	0.08
Visceral fat	–	–	–	–	1.78 [1.08, 2.93]	0.02

^a^Inverse hepatic attenuation in Hounsfield units (HU*-1) visceral fat area (cm^2^).

^b^OR by z-score of inverse hepatic attenuation or visceral fat area (cm^2^).

^c^Adjusted for age, gender, study site.

^d^Model 1 + adjustment for BMI (kg/m^2^), energy intake (kCal/day), Alternative Health Eating Index-2010 (AHEI-2010), physical activity (MET-minutes/week), use of lipid-lowering medications.

In analyses of MET-Liver adjusting for CT-measured intrahepatic fat, the odds of incident diabetes remained significant in the fully adjusted model (OR 1.87 [1.06, 3.27]) (*p* = 0.03). Consistent with this finding, the odds of incident diabetes by MET-Visc adjusted for CT-measured visceral fat area remained robust (OR 2.38 [95% CI 1.34, 4.25] (*p* = 0.003). (Table 2)

After analyses adjusting models for both measured intrahepatic fat and visceral fat area, only visceral fat area remained significantly associated with odds of incident diabetes (OR 1.78 [95% CI 1.08, 2.93]).

When stratifying the at-risk population to those who were normoglycemic at baseline or with impaired fasting glucose, measured adiposity, MET-Liver and MET-VISC had statistically significant associations with incident T2D only in participants who were normoglycemic at baseline (Supplementary Table 2).

## Discussion

In an analysis of South Asian Americans without prevalent diabetes, a metabolite-derived intrahepatic fat score and visceral fat score were both associated with odds of incident type 2 diabetes at five years. These associations remained significant after adjustment for BMI, and also after adjusting for measured adiposity, suggesting that the metabolite-derived score may capture shifts in the metabolic environment beyond those represented by measurable liver or visceral fat.

BMI is an inadequate measure of metabolically risky adiposity, especially in South Asian and East Asian populations [8, 22]. Despite lower World Health Organization (WHO) cutpoints for overweight and obesity, metabolic abnormalities abound in “normal” weight people [8]. In an analysis of the MASALA Study as compared with Black, White, Hispanic and Chinese American participants of the MESA Study, South Asians with a BMI of 19.6 kg/m^2^ had equivalent metabolic abnormalities to people of other race or ethnic groups with much higher BMI values [1]. It is well-known that abdominal obesity is linked with metabolic dysfunction. Intrahepatic fat is associated with metabolic dyslipidemia [5] and insulin resistance [4], and diabetes [23]. Visceral fat is also associated with type 2 diabetes [24] and gestational diabetes [25] more strongly than overall adiposity.

In well-adapted physiological states, excess energy is captured by subcutaneous fat, which expands to store the energy and maintain balance [26]. When excess energy exceeds the capacity of subcutaneous fat to accommodate and store it, there is spillover in the form of free fatty acids which then circulate to house themselves in liver and visceral adipose tissue [24]. In this disrupted metabolic state, there is decreased glucose uptake by skeletal muscles, increased lipolysis, gluconeogenesis and decreased insulin secretion over time [26]. The metabolites identified in this study are likely reflections of these disrupted metabolic processes, representing the etiology behind the associations of ectopic adiposity before clinically-measurable glycemic dysregulation is present. Capturing these initial signals of disrupted processes before clinically relevant aberrations in glycemic control are present, or even before these ectopic fat stores may be measured radiographically, may be a means of using metabolites as biomarkers for intrahepatic fat and visceral adiposity as early biomarkers of T2D risk.

The metabolite score identified in this study uses LC-MS derived circulating metabolites to represent adiposity. Prior work has shown that metabolite-derived scores may have higher correlation with disease prevalence and incidence than the risk factors they represent, such as the link between diet and cardiovascular disease [21]. This may be in part due to the exposure itself – diet is difficult to measure, often determined by self-report, which is subject to some bias. Additionally, given the wide range of metabolites included in our score, these metabolites likely reflect numerous metabolic processes that are affected by metabolically active fat beyond those captured by the presence of the fat itself. Ectopic fat, specifically intrahepatic fat and visceral adiposity, and impaired glycemia likely have bidirectional effects once present; the presence of each contributes to exacerbation of its counterpart [27]. Therefore, the identification of metabolically active adiposity before the presence of glycemic dysfunction may allow for more timely intense lifestyle intervention, including weight management, improvement in diet quality and an increase in moderate and vigorous intensity exercise.

Several metabolites, notably ceramides and sphingolipids, may directly affect and be affected by intrahepatic fat. SulfoHexCer(d18:2/24:1), in particular, had high coefficients of association with both intrahepatic fat and visceral fat in these analyses. Ceramides may be especially important as links between intrahepatic fat and metabolic dysregulation, as indicators of lipid excess and impairment of insulin signaling pathways [28–30]. Saturated fat intake has been related to increased intrahepatic fat and also with increased circulating ceramides [31, 32]. The amino acid proline, previously associated with prevalent and incident T2D in other cohorts, was also highly represented in metabolite scores for both visceral adiposity and intrahepatic fat [33, 34].

Conversely, alpha and beta carotene had strong negative coefficients representing inverse associations with each type of fat in the elastic net regression. These may be representative of fruit and vegetable intake and healthful diet intake overall, which has lower associations with intrahepatic fat [35] and diabetes [36, 37] in epidemiologic studies. In prior NHANES analyses, low serum antioxidant concentration, including alpha- and beta-carotene status, was associated with higher rates of metabolic syndrome [38]. Overall, the presence of these metabolites within the risk score reflects risk factors for metabolic dysregulation. In the future, measurement of metabolite risk scores may serve as a means to monitor the effects of lifestyle changes on ectopic adiposity.

The directionality of association between metabolically active adiposity and glycemic dysregulation is unclear, and the results of our analysis support the possibility that ectopic adiposity may develop prior to clinically notable glycemic change. In supplemental analyses stratifying for glycemia, we found that both adiposity and the metabolite scores for adiposity were associated with incident T2D in those who were normoglycemic at baseline, but not in those with impaired fasting glucose. This further suggests that the presence of intrahepatic fat and visceral adiposity may be an early indicator of metabolic dysfunction, however once impaired glycemia, once present, is the main driver of risk for T2D. Therefore, these associated metabolite signatures, may serve as a biomarker for metabolic dysfunction prior to the evidence of impaired glycemia. These analyses may be affected by sample size, as there were fewer participants with impaired fasting glucose at baseline. Still, our findings suggest that the derived metabolite risk scores may assist in determining risk for type 2 diabetes even before fasting glucose is elevated.

Our analysis has several strengths: a large, well-characterized South Asian population in America with uniquely high risks for type 2 diabetes with robust clinical, demographic, adipose tissue and metabolomics data and the benefit of longitudinal follow-up of metabolic change. Limitations to our analysis are present, including metabolomics measured at one time point and a population already aged at least 40 years an entrance into the cohort; we could not identify early-life risk for adiposity. We did not have time-to-event data as there was data from only one follow-up available. The metabolite score was calculated based on observed data, then used in a regression model which treated it as observed data, thereby underestimating the variance in the calculated score as compared with the observed data. Therefore, we cannot directly compare strength of association between the observed and calculated odds ratios.

Still, since the majority of T2D screening is based on risk stratification by BMI, our findings suggest that characterizing adiposity and associated metabolic changes in a population with lower rates of overweight and obesity may aid in defining T2D risk. As these relationships remained strong despite adjustment for BMI, use of a metabolite risk score as a biomarker of visceral and intrahepatic fat can signal elevated risk for T2D regardless of the presence of BMI-defined overweight or obesity.

## Conclusion

In a population of South Asians in the United States, metabolite risk scores representative of visceral fat and intrahepatic fat were associated with incident diabetes. Future work including the metabolites most representative of these adipose fat depots as measures of risk may help target prevention for future type 2 diabetes, especially in those populations without traditional risk factors of overweight or obesity.

### Supplementary information

Suppemental Material

## Data Availability

Data described in the manuscript, code book, and analytic code will be made available upon request pending request to MASALA Study Steering Committee for reasons of participant confidentiality
