# Supplementary material for: Adipose tissue-derived metabolite risk scores and risk for type 2 diabetes in South Asians
Source: Int J Obes (Lond). 2024 Jan 20;48(5):668–73. doi: 10.1038/s41366-023-01457-4 (PMC11058083; doi:10.1038/s41366-023-01457-4)
Supplement: Supplementary file 1 — Suppemental Material [file 41366_2023_1457_MOESM1_ESM.docx]

**Supplemental Figure Legend:**

Flow diagram of participant inclusion. Participants from the MASALA cohort were excluded if they were missing baseline metabolomics data, computed tomography measurements of visceral and intrahepatic fat, and prevalent diabetes.

**Supplemental Figure:**

**Supplemental Table 1: Metabolites Selected by Elastic Net Regularized Regression by Type of Adiposity and their Coefficients**

| **Intrahepatic Fat** |  |  | **Visceral Fat** |  |
| --- | --- | --- | --- | --- |
| zetacarotene | 0.69 |  | alphacarotenebetacarotene | -3.57 |
| lpi16100 | -0.64 |  | sulfohexcerd182220 | -3.36 |
| alphacarotenebetacarotene | 0.59 |  | sulfohexcerd182241 | -2.86 |
| sulfohexcerd181241 | 0.58 |  | proline | 2.60 |
| peo161225andorpep160225 | -0.51 |  | sulfohexcerd181241 | -2.52 |
| sulfohexcerd181180 | 0.48 |  | cert180241 | 2.47 |
| pc203204 | 0.45 |  | methylnicotinamide | -2.45 |
| lpi18200 | -0.42 |  | sulfohexcerd181181 | -2.44 |
| nacetyldmannosamine | -0.39 |  | pantothenate | 2.16 |
| fa160oh | -0.39 |  | trimethylaminoacetone | 2.14 |
| pc180203 | -0.39 |  | niacinamide | -2.07 |
| deltatocopherol | -0.37 |  | peo161204andorpep160204 | 2.01 |
| dg160160 | -0.35 |  | cholesterolsulfate | 1.98 |
| fa244 | -0.33 |  | lpc22000 | -1.91 |
| sulfohexcerd181181 | 0.30 |  | histidine | 1.90 |
| pc140226 | 0.30 |  | smd182140 | 1.89 |
| lpi16000 | -0.30 |  | n1acetylspermidine | 1.86 |
| lpc00161 | -0.29 |  | pe160203pe181182pe180183 | -1.85 |
| sulfohexcerd182241 | 0.29 |  | anhydroluteini | -1.84 |
| sulfohexcerd182220 | 0.29 |  | lpc22500 | -1.81 |
| luteine | 0.29 |  | pc203204 | -1.80 |
| proline | -0.28 |  | pc182182 | -1.79 |
| lpco26100 | 0.28 |  | tetracosenoylcarncar241 | 1.75 |
| methylnicotinamide | 0.27 |  | pa160181 | -1.74 |
| ce203 | -0.26 |  | tg582 | 1.74 |
| cerd180220 | -0.25 |  | pe181182 | -1.72 |
| car240 | -0.25 |  | car140oh | 1.69 |
| ce183 | 0.24 |  | nndimethylglycine | 1.68 |
| pe201204 | 0.24 |  | pco160182 | -1.66 |
| betaine | 0.24 |  | sulfohexcerd181241oh | 1.61 |
| pc321pc140181andpc160161 | -0.23 |  | smd182160 | 1.60 |
| fa120 | 0.23 |  | pco161182andorpcp160182 | -1.60 |
| hydroxybutyrylcarnitinec40oh | -0.23 |  | nacetyldmannosamine | 1.57 |
| cerd180240 | -0.22 |  | pc160181 | -1.54 |
| fa140 | 0.22 |  | lpc20300_2 | -1.53 |
| cerd340cerd180160cerd160180 | -0.22 |  | ce183 | -1.53 |
| lpc22200 | 0.22 |  | glutamine | -1.53 |
| tg443 | -0.22 |  | pe180204 | -1.51 |
| lpg18200 | -0.22 |  | fa225_2 | -1.49 |
| pco160182 | 0.21 |  | sulfohexcerd181180 | -1.47 |
| tg501 | -0.21 |  | methylguanine | 1.46 |
| v7 | -0.21 |  | lpg18200 | 1.46 |
| dg180160 | -0.21 |  | sulfo2hexcerd421 | 1.40 |
| caffeine | -0.20 |  | tg561 | 1.40 |
| carnitine | 0.20 |  | pe160204 | -1.39 |
| lpco24100 | 0.20 |  | pc140226 | -1.39 |
| lpc00225 | 0.20 |  | tg562 | 1.33 |
| lpc20300_2 | 0.20 |  | fa241 | 1.31 |
| fa240 | 0.20 |  | fa244 | 1.31 |
| sulfohexcerd182240oh | -0.20 |  | lpc18200 | -1.30 |
| biliverdin | -0.19 |  | tg581 | 1.29 |
| pc140204 | 0.19 |  | smd301smd161140smd181120 | 1.28 |
| pc180224 | -0.19 |  | lpi16100 | 1.22 |
| pe180204 | 0.19 |  | peo161182andorpep160182 | 1.20 |
| pe160203pe181182pe180183 | 0.19 |  | lpc20100 | 1.19 |
| sulfohexcerd181240oh | -0.19 |  | fa260 | -1.19 |
| dg342 | -0.19 |  | sulfo2hexcerd401 | 1.19 |
| tg589_1 | -0.19 |  | fa100 | -1.18 |
| pco161182andorpcp160182 | 0.19 |  | car201 | 1.18 |
| peo161226andorpep160226 | -0.19 |  | taurine | 1.18 |
| lpc22000 | 0.19 |  | caffeine | 1.17 |
| fa225_2 | 0.18 |  | fa184 | -1.17 |
| lpco20000 | 0.18 |  | pc181203 | -1.16 |
| lpi20300 | -0.18 |  | sulfo2hexcerd341 | 1.15 |
| creatinine | -0.17 |  | lpc00182 | -1.14 |
| fa162 | 0.17 |  | tg5810_2 | 1.13 |
| cerd182240 | 0.17 |  | lpc00181 | -1.13 |
| pco241224andorpcp240224 | -0.17 |  | tg602 | 1.13 |
| fa171 | 0.17 |  | sulfohexcerd182220oh | -1.12 |
| fa241 | -0.17 |  | pco160160 | 1.11 |
| tg547_3 | -0.17 |  | lpc19000 | -1.10 |
| dg160181 | -0.16 |  | methyl2piperidinecarboxylate | 1.09 |
| pco221204andorpcp220204 | 0.16 |  | peo161226andorpep160226 | 1.08 |
| pc180181 | -0.16 |  | pc160160 | 1.08 |
| pe160204 | 0.16 |  | deltatocopherol | 1.06 |
| peo181224andorpep180224 | -0.16 |  | smd321smd161160smd181140 | 1.05 |
| cerd423cerd182241cerd181242 | 0.16 |  | fa201 | 1.05 |
| smd171241 | 0.16 |  | lpc22600 | -1.05 |
| tg533 | 0.16 |  | lpa20400 | 1.05 |
| peo161182andorpep160182 | -0.15 |  | zetacarotene | -1.03 |
| tg5810_1 | -0.15 |  | lpe00180 | 1.02 |
| cert180241 | -0.15 |  | smd191241 | 1.00 |
| fa260 | 0.15 |  | pc180226 | 0.99 |
| tg5810_2 | -0.15 |  | peo181181andorpep180181 | 0.99 |
| tetracosenoylcarncar241 | -0.14 |  | lpe18100 | 0.98 |
| cerd191240 | 0.14 |  | betaine | 0.98 |
| lpc18200 | 0.14 |  | car260 | -0.97 |
| fa161 | 0.14 |  | hydroxybutyrylcarnitinec40oh | 0.96 |
| tg547_4 | -0.14 |  | tg567_1 | -0.96 |
| lpco18000 | 0.14 |  | biliverdin | 0.95 |
| lpc18300_1 | 0.14 |  | pco221204andorpcp220204 | 0.94 |
| pc180180 | 0.14 |  | ce182 | -0.94 |
| sphingosine1phosphate | -0.14 |  | tg460 | 0.94 |
| tg565 | 0.13 |  | pc180180 | -0.93 |
| car140oh | -0.13 |  | pco180160 | 0.90 |
| lpc22500 | 0.13 |  | sulfo2hexcerd422 | 0.89 |
| pc160203 | -0.13 |  | tg604_2 | -0.88 |
| peo160226 | -0.13 |  | pc180225 | -0.87 |
| glutamine | 0.13 |  | peo182182andorpep181182 | 0.87 |
| nndimethylglycine | 0.13 |  | dg160181 | 0.87 |
| lpa20400 | -0.13 |  | lpi16000 | 0.87 |
| tg588 | -0.13 |  | lpc20000 | -0.86 |
| pco180204 | 0.13 |  | lpc00203 | -0.85 |
| tg442 | -0.13 |  | fa240 | -0.85 |
| cerd161200 | -0.13 |  | pc180203 | 0.84 |
| smd182241 | 0.13 |  | smd161200 | 0.84 |
| ce181 | 0.12 |  | dg160160 | 0.83 |
| tg602 | 0.12 |  | creatinine | 0.83 |
| ce226 | 0.12 |  | lpc00226 | -0.82 |
| tg568 | -0.12 |  | napeo181204180napep18020418 | -0.82 |
| zeaxanthin | -0.12 |  | pc160183 | -0.82 |
| smd181240 | -0.12 |  | car180 | -0.81 |
| lpc00183 | 0.12 |  | lpc00225 | -0.81 |
| lpc22600 | 0.11 |  | car100oh | -0.81 |
| tg581 | -0.11 |  | tg541 | 0.80 |
| cerd181180 | -0.11 |  | tg566_1 | -0.79 |
| tg534 | 0.11 |  | pco160203 | -0.79 |
| tg567_3 | -0.11 |  | luteine | -0.78 |
| tg587_1 | -0.11 |  | smd171160 | 0.78 |
| lpi22600 | -0.11 |  | ce205 | -0.78 |
| tg502 | -0.11 |  | cerd182180 | -0.78 |
| lpi00203 | -0.11 |  | dg321 | 0.78 |
| lpc00181 | 0.11 |  | pc331pc150181pc160171 | -0.77 |
| tg604_1 | -0.11 |  | pe180203 | -0.77 |
| lpc16100 | -0.11 |  | tg501 | 0.77 |
| pc180182 | -0.10 |  | fa160oh | 0.76 |
| tg5811 | -0.10 |  | tg461 | 0.74 |
| smd180160 | -0.10 |  | lpe20400 | 0.73 |
| pc340180160andpc160180 | 0.10 |  | car161 | 0.73 |
| lpc17100 | 0.10 |  | lpi18200 | 0.73 |
| hexadecadienoylcarncar162 | -0.10 |  | lpc20300_1 | -0.72 |
| dodecenoylcarnitine121 | -0.10 |  | smd161220 | 0.72 |
| smd182220 | 0.10 |  | tg565 | -0.71 |
| tg526_2 | 0.10 |  | dg342 | 0.71 |
| car161 | -0.10 |  | sulfohexcerd181220oh | 0.71 |
| hexadecenoylcarnitinec161 | -0.10 |  | laccerd181241 | -0.70 |
| pc160226 | 0.09 |  | hexcerd181230 | -0.70 |
| pc160224 | -0.09 |  | smd161241 | 0.70 |
| fa100 | -0.09 |  | sulfohexcerd181160oh | 0.68 |
| peo161204andorpep160204 | -0.09 |  | trimethylaminenoxide | 0.64 |
| lpc19000 | 0.09 |  | cerd181250 | -0.63 |
| peo181181andorpep180181 | -0.09 |  | fa140 | -0.63 |
| peo181182andorpep180182 | -0.09 |  | cerd340cerd180160cerd160180 | 0.62 |
| lpi18100 | -0.09 |  | tg546_2 | 0.61 |
| pipecolatenmethylproline | 0.09 |  | tg589_2 | 0.61 |
| smd351smd171180andsmd181170 | 0.08 |  | pc403 | -0.61 |
| tg460 | -0.08 |  | pseudouridine | 0.59 |
| tg608 | -0.08 |  | tg531 | 0.58 |
| ce204 | 0.08 |  | tg527 | 0.58 |
| ce205 | 0.08 |  | tg553 | -0.58 |
| pc182182 | 0.08 |  | car202 | 0.58 |
| cerd182180 | -0.08 |  | tg521 | 0.58 |
| car201 | -0.08 |  | hexcerd181200 | -0.57 |
| tg589_2 | -0.08 |  | cerd182240 | -0.56 |
| tg541 | -0.08 |  | tg502 | 0.56 |
| lpc00202 | -0.08 |  | car142 | -0.56 |
| tg505_2 | 0.08 |  | bilirubin | -0.54 |
| lpc00226 | 0.08 |  | pc180204 | 0.53 |
| tg525_3 | -0.08 |  | fa181oh | 0.53 |
| lpc00204 | 0.08 |  | tg564 | -0.53 |
| trigonelline | 0.08 |  | lpe18200 | 0.53 |
| tg532 | 0.07 |  | retinol | -0.53 |
| pe180160 | 0.07 |  | tg542 | 0.52 |
| tg525_2 | 0.07 |  | tg554 | -0.52 |
| tg603 | 0.07 |  | trigonelline | -0.52 |
| tg546_2 | -0.07 |  | smd182230 | -0.52 |
| smd191241 | 0.07 |  | pc140204 | -0.51 |
| paraxanthine | -0.07 |  | cerd161200 | -0.51 |
| pantothenate | 0.07 |  | tg547_3 | 0.51 |
| pc160183 | -0.07 |  | tg490 | -0.50 |
| methyl2piperidinecarboxylate | 0.07 |  | sulfo2hexcerd411 | 0.50 |
| tg490 | 0.07 |  | smd182240 | -0.50 |
| propionylcarnitine | 0.07 |  | smd191180 | 0.50 |
| lpc00182 | 0.07 |  | dg180160 | -0.49 |
| car204 | 0.07 |  | car261 | -0.49 |
| tg535 | 0.07 |  | cortisol | -0.49 |
| car180dc | -0.07 |  | tg526_1 | 0.49 |
| lpco24000 | 0.07 |  | pco220203 | 0.49 |
| car181dc | -0.06 |  | pc160150 | -0.49 |
| tg567_1 | -0.06 |  | napeo181204160napep18020416 | -0.48 |
| tg484 | 0.06 |  | tg587_2 | -0.47 |
| cerd181250 | 0.06 |  | tg548_2 | 0.46 |
| pc331pc150181pc160171 | 0.06 |  | lpi20400 | 0.46 |
| dg180181 | -0.06 |  | lpe00204 | 0.46 |
| pco241204andorpcp240204 | -0.06 |  | smd181201 | 0.46 |
| tg562 | -0.06 |  | pa160182 | -0.44 |
| sulfo2hexcerd422 | 0.06 |  | peo181182andorpep180182 | 0.44 |
| tg421 | -0.06 |  | sphingosine1phosphate | 0.44 |
| laccerd181241 | 0.06 |  | sulfohexcerd182160oh | 0.44 |
| peo182204andorpep181204 | -0.06 |  | fa183_1 | -0.43 |
| lpe00182 | -0.05 |  | lpcp18000 | -0.41 |
| octenoylcarnitinecar81 | -0.05 |  | peo160226 | 0.41 |
| pa160181 | -0.05 |  | pao181204andorpap180204 | 0.40 |
| tg566_1 | -0.05 |  | smd191160 | 0.40 |
| pao181204andorpap180204 | -0.05 |  | pc160224 | -0.40 |
| pc160204_2 | 0.05 |  | cerd181261 | 0.39 |
| tg569 | -0.05 |  | pco241204andorpcp240204 | 0.38 |
| pseudouridine | 0.05 |  | peo182204andorpep181204 | 0.38 |
| pe180203 | 0.05 |  | tg504 | 0.37 |
| smd161241 | 0.05 |  | tg481 | 0.37 |
| pc403 | -0.05 |  | laccerd423 | -0.37 |
| laccerd181160 | 0.05 |  | tg505_3 | 0.36 |
| lpe18000 | 0.05 |  | zeaxanthin | -0.36 |
| car120 | -0.05 |  | cerd161220 | 0.36 |
| pe181182 | 0.05 |  | ce181 | -0.36 |
| anhydroluteini | 0.05 |  | sulfohexcerd181180oh | 0.36 |
| n1methyl2pyridone5carboxamide | -0.04 |  | smd182180 | 0.35 |
| cerd182220 | 0.04 |  | pco241225andorpcp240225 | -0.35 |
| tg567_2 | -0.04 |  | pc160204_1 | -0.34 |
| sulfohexcerd181220oh | -0.04 |  | tg525_1 | 0.34 |
| bilirubin | -0.04 |  | pe160182 | -0.34 |
| car181 | -0.04 |  | hexanoylcarnitinec60 | -0.34 |
| car261 | -0.04 |  | cerd191240 | 0.34 |
| taurine | -0.04 |  | dg180181 | 0.33 |
| pco180224 | 0.04 |  | ce204 | -0.33 |
| car140 | -0.03 |  | car100 | -0.33 |
| cerd181200 | -0.03 |  | carnitine | -0.33 |
| pco220182 | 0.03 |  | lpe18000 | -0.32 |
| fa201 | -0.03 |  | peo201204andorpep200204 | 0.32 |
| lpc00203 | -0.03 |  | tg524 | 0.31 |
| smd181230 | -0.03 |  | cerd161240 | 0.31 |
| tg548_1 | 0.03 |  | pc160170 | -0.31 |
| octenoylcarnitinec81 | -0.03 |  | tg604_1 | 0.31 |
| isovalerylcarnitine | -0.03 |  | tg547_4 | 0.30 |
| tg493 | 0.03 |  | fa204 | 0.30 |
| lpe18100 | -0.03 |  | napeo181181160napep18018116 | -0.29 |
| pco181226andorpcp180226 | 0.03 |  | lpc18300_2 | -0.28 |
| lpe18200 | -0.03 |  | tg552 | 0.28 |
| pco220203 | -0.03 |  | tg5810_1 | 0.27 |
| lpcp18000 | 0.03 |  | cerd201240 | 0.27 |
| dg321 | -0.03 |  | peo201182andorpep200182 | 0.26 |
| car102 | -0.03 |  | cerd181200 | -0.25 |
| cholesterol | 0.03 |  | tg525_3 | 0.25 |
| fa184 | 0.03 |  | ce203 | 0.25 |
| fa181 | 0.02 |  | lpc22400 | -0.24 |
| car260 | -0.02 |  | tg505_2 | -0.23 |
| tg521 | -0.02 |  | lacetylcarnitine | -0.23 |
| smd181201 | 0.02 |  | fa161 | -0.23 |
| trimethylaminenoxide | 0.02 |  | hexadecadienoylcarncar162 | 0.23 |
| cerd181261 | 0.02 |  | smd181180 | -0.22 |
| lpc20400 | 0.02 |  | hexadecenoylcarnitinec161 | 0.22 |
| cerd181241 | 0.02 |  | tg587_1 | 0.22 |
| smd182230 | 0.02 |  | pco241203andorpcp240203 | 0.20 |
| cortisol | 0.02 |  | fa224 | 0.20 |
| lpc20000 | 0.02 |  | tg535 | 0.20 |
| cerd181240 | 0.02 |  | pe180225 | -0.19 |
| sulfohexcerd182160oh | 0.01 |  | lpco18000 | 0.19 |
| hydroxypalmitoleoylcarncar1 | 0.01 |  | tg462 | 0.19 |
| tg563 | -0.01 |  | lpe00182 | 0.18 |
| smd182140 | 0.01 |  | pc160226 | -0.18 |
| car203 | -0.01 |  | cerd161241 | 0.18 |
| smd182240 | 0.01 |  | car102 | 0.18 |
| lpc20300_1 | -0.01 |  | car240 | 0.18 |
| fa182 | 0.01 |  | arachidylcarnitinecar200 | 0.17 |
| methyladenosine | -0.01 |  | pco261204andorpcp260204 | 0.17 |
| car182 | -0.01 |  | v219 | 0.17 |
| creatine | -0.01 |  | tg568 | 0.16 |
| smd191160 | 0.01 |  | tg482 | 0.16 |
| tg587_2 | 0.00 |  | tg491 | 0.16 |
| car120oh | 0.00 |  | tg546_1 | 0.16 |
| car142 | 0.00 |  | laccerd181160 | 0.16 |
| smd181180 | 0.00 |  | fa202 | 0.16 |
| pco161226andorpcp160226 | 0.00 |  | cerd182220 | -0.15 |
| lpc20500 | 0.00 |  | creatine | -0.15 |
| car202 | 0.00 |  | pe180182 | -0.14 |
| car141 | 0.00 |  | asymmetricdimethylarginine | -0.14 |
| pco160150 | 0.00 |  | hydroxypalmitoleoylcarncar1 | -0.14 |
| hexcerd181240 | 0.00 |  | pc140182 | -0.14 |
| _cons | 0.00 |  | tg443 | -0.14 |
|  |  |  | sulfohexcerd182240oh | -0.13 |
|  |  |  | tg5811 | 0.13 |
|  |  |  | octenoylcarnitinecar81 | -0.13 |
|  |  |  | lpc00202 | 0.13 |
|  |  |  | car120 | -0.12 |
|  |  |  | laccerd181240 | -0.12 |
|  |  |  | tg567_3 | -0.11 |
|  |  |  | cerd181241 | 0.11 |
|  |  |  | pco220182 | -0.11 |
|  |  |  | car180dc | -0.10 |
|  |  |  | octenoylcarnitinec81 | -0.10 |
|  |  |  | sulfohexcerd181240oh | 0.10 |
|  |  |  | tg563 | 0.10 |
|  |  |  | pco242204andorpcp241204 | 0.09 |
|  |  |  | tg471 | 0.09 |
|  |  |  | pe180224 | -0.09 |
|  |  |  | pco160150 | 0.08 |
|  |  |  | smd171241 | 0.08 |
|  |  |  | tg421 | -0.08 |
|  |  |  | car141 | -0.07 |
|  |  |  | lpe22600 | 0.06 |
|  |  |  | tg547_1 | 0.06 |
|  |  |  | tg526_3 | 0.06 |
|  |  |  | dodecenoylcarnitine121 | 0.06 |
|  |  |  | n1methyl2pyridone5carboxamide | 0.05 |
|  |  |  | tg566_2 | -0.05 |
|  |  |  | tg589_1 | 0.05 |
|  |  |  | tg608 | 0.05 |
|  |  |  | tg492 | 0.04 |
|  |  |  | lpi20300 | 0.04 |
|  |  |  | tg505_1 | 0.04 |
|  |  |  | cerd411cerd181230andcerd171240 | -0.04 |
|  |  |  | peo161225andorpep160225 | 0.03 |
|  |  |  | cerd181180 | -0.03 |
|  |  |  | tg514 | 0.03 |
|  |  |  | tg463 | 0.03 |
|  |  |  | fa120 | -0.03 |
|  |  |  | lpi18100 | 0.01 |
|  |  |  | pc180182 | 0.01 |
|  |  |  | dg182181 | 0.01 |
|  |  |  | tg484 | 0.01 |
|  |  |  | smd180160 | 0.01 |
|  |  |  | pc180181 | -0.01 |
|  |  |  | pc160182 | -0.01 |

**Supplemental Table 2: Odds of Incident Diabetes At 5 Years by Measured and Predicted Values for Hepatic Attenuation and Visceral Fat Area^a^ by Baseline Glycemia**

|  | Odds^b^ of Incident Diabetes, Odds Ratio [95% CI] | | | |
| --- | --- | --- | --- | --- |
|  | Normoglycemic^c^ (n=389) | | Impaired fasting glucose^c^ (n=150) | |
| Incident diabetes (n) | 26 | | 21 | |
| Hepatic fat, measured by CT or metabolite score | | | | |
|  | OR [95% CI] | P value | OR [95% CI] | P value |
| Intrahepatic fat | 1.86 (1.26, 2.74) | 1.86e-03 | 1.03 (0.63, 1.67) | 0.91 |
| MET-Liver | 2.30 (1.33, 3.95) | 2.67e-03 | 1.36 (0.77, 2.39) | 0.29 |
| Visceral fat, measured by CT or metabolite score | | | | |
| Visceral fat | 2.05 (1.20, 3.51) | 0.01 | 1.74 (0.75, 4.07) | 0.20 |
| MET-Visc | 2.58 (1.27, 5.22) | 0.01 | 2.04 (0.81, 5.13) | 0.13 |

^a^ Inverse hepatic fat attenuation in Hounsfield units (HU); visceral fat area (cm^2^)

^b^ OR by z-score of inverse hepatic attenuation or visceral fat area

^c^ Adjusted for age, gender, study site, body mass index (kg/m^2^), energy intake (kCal/day), Alternative Health Eating Index-2010 (AHEI-2010), physical activity (MET-minutes/week), use of cholesterol-lowering medications
